# Natural Bioactive Molecules: An Alternative Approach to the Treatment and Control of COVID-19

**DOI:** 10.3390/ijms222312638

**Published:** 2021-11-23

**Authors:** Fahadul Islam, Shabana Bibi, Atkia Farzana Khan Meem, Md. Mohaimenul Islam, Md. Saidur Rahaman, Sristy Bepary, Md. Mizanur Rahman, Md. Mominur Rahman, Amin Elzaki, Samih Kajoak, Hamid Osman, Mohamed ElSamani, Mayeen Uddin Khandaker, Abubakr M. Idris, Talha Bin Emran

**Affiliations:** 1 Department of Pharmacy, Daffodil International University, Dhaka 1207, Bangladesh; fahadulislamdiu@gmail.com (F.I.); meematkia10@gmail.com (A.F.K.M.); mifoysal1569@gmail.com (M.M.I.); saidur29-1299@diu.edu.bd (M.S.R.); sristy29-917@diu.edu.bd (S.B.); mizanur.ph@diu.edu.bd (M.M.R.); mominur.ph@gmail.com (M.M.R.); 2 Yunnan Herbal Laboratory, College of Ecology and Environmental Sciences, Yunnan University, Kunming 650091, China; shabana_bibi@ynu.edu.cn; 3 International Joint Research Center for Sustainable Utilization of Cordyceps Bioresources in China and Southeast Asia, Yunnan University, Kunming 650091, China; 4 Department of Radiological Sciences, College of Applied Medical Sciences, Taif University, Taif 21944, Saudi Arabia; a.zaki@tu.edu.sa (A.E.); s.kajoak@tu.edu.sa (S.K.); ha.osman@tu.edu.sa (H.O.); m.samani@tu.edu.sa (M.E.); 5 Centre for Applied Physics and Radiation Technologies, School of Engineering and Technology, Sunway University, Bandar Sunway 47500, Selangor, Malaysia; mayeenk@sunway.edu.my; 6 Department of Chemistry, College of Science, King Khalid University, Abha 62529, Saudi Arabia; abubakridris@hotmail.com; 7 Research Center for Advanced Materials Science (RCAMS), King Khalid University, Abha 62529, Saudi Arabia; 8 Department of Pharmacy, BGC Trust University Bangladesh, Chittagong 4381, Bangladesh

**Keywords:** bioactive molecules, alkaloids, flavonoids, terpenoids, coronavirus, COVID-19, SARS-CoV-2

## Abstract

Several coronaviruses (CoVs) have been associated with serious health hazards in recent decades, resulting in the deaths of thousands around the globe. The recent coronavirus pandemic has emphasized the importance of discovering novel and effective antiviral medicines as quickly as possible to prevent more loss of human lives. Positive-sense RNA viruses with group spikes protruding from their surfaces and an abnormally large RNA genome enclose CoVs. CoVs have already been related to a range of respiratory infectious diseases possibly fatal to humans, such as MERS, SARS, and the current COVID-19 outbreak. As a result, effective prevention, treatment, and medications against human coronavirus (HCoV) is urgently needed. In recent years, many natural substances have been discovered with a variety of biological significance, including antiviral properties. Throughout this work, we reviewed a wide range of natural substances that interrupt the life cycles for MERS and SARS, as well as their potential application in the treatment of COVID-19.

## 1. Introduction

Viral infections are currently considered as a public health concern. Viruses are non-living submicroscopic agents that cause a variety of human ailments. They are often composed of RNA or DNA strains. Measles, human immunodeficiency virus (HIV), influenza, herpes simplex virus, and dengue virus are the most well-known viral diseases [1]. Coronaviruses (CoVs) are members of the *Coronaviridae* family and look like spiky rings under an electron microscope. Their surface is covered in spikes that aid in the attack and binding of living cells [2]. Among the many coronaviruses, notably, coronavirus disease 2019 (COVID-19), first recorded in China in December 2019, is caused by severe acute respiratory syndrome coronavirus 2 (SARS-CoV-2). It has already spread across the world, infecting millions of people [3,4]. COVID-19 has prevailed almost across the whole world and many countries’ healthcare services have now reached their breaking point. The disease spreads through respiratory droplets and direct contact, and city and community disinfection have not proven to be successful in controlling this disease [5]. On 11 March 2020, the World Health Organization (WHO) classified the COVID-19 breakout a pandemic [6]. COVID-19 has affected over 219 countries and territories throughout the world, resulting in around 190 million reported cases and over four million fatalities [7]. Despite the findings of this viral entity and a greater understanding of its mechanism of transmission, SARS-CoV-2 continues to infect hundreds of thousands of individuals every day throughout the world. The new coronavirus, SARS-CoV-2, is closely linked to bat coronavirus, with over 88% nucleotide sequence identity, according to phylogenic research. As a result, it has been categorized as a severe acute respiratory syndrome coronavirus (SARS-CoV) (80% nucleotide similarity), such as the virus that produced the severe acute respiratory syndrome (SARS) epidemic in Guangdong Province, China, in 2002 and 2003, and similar to the Middle East respiratory syndrome coronavirus (MERS-CoV) (% nucleotide similarity) that produced the Middle East respiratory syndrome (MERS) epidemic in the Arabian Peninsula in 2012 [8].

In folk medicine, natural products and their derivatives are utilized to cure a variety of diseases, such as viral infections. Throughout the field of nutraceuticals, herbal drugs have a variety of applications [9,10,11]. By limiting virus replication, certain natural compounds have been shown to have antiviral characteristics. Aside from plant-derived chemicals, some marine natural products and biotechnologically generated compounds have also been demonstrated to have antiviral properties against various viruses [12,13,14,15,16,17]. Nature has a wide reservoir of compounds that may be used to research and produce medications for a variety of ailments, including viral infections. A large variety of herbal medications and their ingredients have proven antiviral efficacy in the past [18,19,20]. There is a scarcity of studies into the creation of anti-coronavirus therapies based on natural compounds. These medicines are vital not only for combating coronavirus, but also for preventing viral infection. Based on previous debates, the goal of this review is to assess the current situation of natural compounds and/or their compounds working against different species of coronavirus.

## 2. Pathogenesis of COVID-19

COVID-19, an extremely lethal disease produced by the SARS-CoV-2 virus, is a major public health concern around the world. In humans, the SARS-CoV-2 virus enters the lower respiratory tract and causes pneumonia [21]. In the earlier stages, it shows only a little respiratory dysfunction, but later move to a fatal respiratory dysfunction syndrome and hyper inflammation. The SARS-CoV and MERS-CoV diseases appear to have similar immunopathogenic features, where acute respiratory distress syndrome (ARDS) is the major cause of death in most of the infectious diseases, including COVID-19 disease. One of the most noticeable aspects is the cytokine storm, which is an unregulated systemic inflammatory process induced by immune cells producing pro-inflammatory cytokines and chemokines [22,23]. Increased blood stages of chemokines and cytokines, such as basic FGF2, GCSF, MIP1, PDGFB, TNF, GMCSF, IL1, IL9, IL10, IL1RA, IL7, IFN, IP10, IL8, FN, IP10, MCP1, MIP1, and VEGFA, are found in COVID-19 patients [24]. Identical to SARS and MERS-CoV infections, a robust inflammatory immune reaction is induced in serious forms of SARS-CoV-2 disease, which leads to organ failure, ARDS, and ultimately death [22]. The cytokine storm harms the lungs and many other internal organs, which is often the leading cause of death in acute COVID-19, including the heart, kidneys, and liver, which results in multiple organ failure [17,25,26,27,28].

## 3. Epidemiology

Transmission by people is the major pathway for getting infected with the SARS-CoV-2 virus, with droplets through sneezing and coughing inhaled via close contact. COVID-19 virus transmission can also occur through contaminated sites or fomites after interaction with the mouth, eye, or nose. Symptomatic patients have a high risk of spreading the virus to others [29]. Asymptomatic patients have less evidence of viral shedding, whereas critically ill patients seem to have higher viral shedding levels [30]. The virus is extremely virulent and was shown to have a lengthy spreading period in China, according to current trends in coronavirus illness epidemiology [31,32,33,34,35,36,37]. In the first coronavirus pandemic in Wuhan, an epidemiological analysis of 425 coronavirus patients was conducted, where 56% of the infected individuals were male, with a median age of 59 years. In total, 86.6% of the affected people were between the ages of 30 and 70 on 11 February 2020. Patients had a total mortality rate of 2.3%, with 80.9% of the recorded cases being moderate. Healthcare professionals accounted for about 3.8% of reported cases in hospitals, with 14.6% of these cases being chronic or serious. Infants infected with the virus were observed in only a few cases (2.1%). The coronavirus was found to have the greatest impact on people above the age of 80, representing 14.8% of all cases. For COVID-19 patients to be classed as chronic, the main elements must be present: a PaO_2_/FiO_2_ higher than 300, oxygen saturated higher than or equal to 93%, respiration larger than or equal to 30 breath/min, and dyspnea, defined as a 50% involvement of the lungs in less than 24 to 48 h. The virus is infectious and at the critical phase in patients with multiple organ dysfunction, leads to septic shock and/or restricted breathing [29,38]. The suggested 14-day initial infection for coronavirus illness is based on the relevant incubation duration for coronaviruses with similar incubation periods after the initial exposure. Incubation takes an average of 5.2 days, with 95% Cl of 4.1 to 7.0, but it can take from 2 to 14 days. Gao et al. also discovered a 9-day average incubation period [39]. Another study revealed that the delta variant’s incubation takes an average of 4 days [40]. Associated infections can be found in roughly 22–33% of infected people, and they may be greater in people with severe illnesses [29].

## 4. Etiology

**Figure 1:**
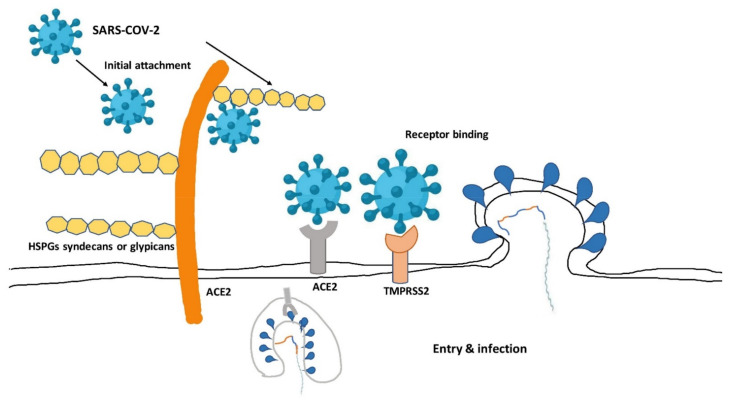
SARS-CoV-2 infection and entrance via a plausible pathway. Using the S protein projecting from the viral particle, SARS-CoV-2 may first engage with the HSPGs on the surface of susceptible cells early in the infection cycle. The virus’s subsequent interaction with the high-affinity entry receptor ACE2 may be facilitated by this initial attachment. By cleaving the S protein at the S1/S2 and/or S2’ sites, the transmembrane protease serine 2 (TMPRSS2) on the host cell surface and other host cell proteases may facilitate viral entry [53]. Adapted with permission from ref. [53]. Copyright 2021 Science China Press.

The virus has an 88% sequencing resemblance to two bat-derived severe acute respiratory syndrome (SARS) coronaviruses, but is more distanced from SARS-CoV [41,42]. Here, as consequence, the virus was dubbed a novel coronavirus in 2019. A coronavirus seems to be a single-stranded, encapsulated ribonucleic acid with surface spikes that are 9 to 12 nm long and mimics the solar corona [43,44,45]. The spike (S) protein interacts with the angiotensin-converting enzyme 2 (ACE2) receptor and facilitates viral entrance into the host cell by mediating fusion of the enveloped and host cell membranes, and the coronavirus genome encodes four key structural protein molecules on the surface [46,47]. Glycosaminoglycans (GAGs) and sialic acid-containing oligosaccharides are examples of such molecules. GAGs are typically found on the cell’s outer surface. They are particularly well suited to function as attachment factors to recruit viruses to the cell surfaces because of their position [48,49,50]. In mammals, heparan sulfate (HS) is one of the most common forms of GAGs. It is a sulfated linear polysaccharide found on the surface of practically all cell types as well as in the extracellular matrix. To produce HS proteoglycans (HSPGs), the HS chains are typically covalently attached as side chains to the core proteins (Figure 1) [51,52].

Many distinct enzymes produce HS in the Golgi apparatus. HS undergoes a number of changes during and after arrangement, including sulfation, acetylation, and epimerization, resulting in glycan structures with a high degree of variation in length, sulfation, and glucuronate/iduronate ratio. In distinct species, organs, tissues, and even at different ages and illness stages, there was significant diversity in the sulfation pattern and degree of HS [54,55]. Many viruses’ attachment to host cells during transmission has been demonstrated to be regulated by the sequence and sulfation pattern of HS. Sialylation patterns of cell surface oligosaccharides showed similar results. The MERS-CoV S protein, for instance, selectively attaches α2,3-linked sialic acids on the cell surface over α2,6-linked sialic acids, and 5-*N*-glycolylation and 7,9-*O*-acetylation of sialic acids impairs their interaction. These data suggest that the distribution of distinct forms of HS/sialylated glycans and viral tropism may have a linkage [56,57,58]. A deeper knowledge of their interaction could involve the development of novel antiviral drugs. Although there is presently inadequate evidence on the viral tropism of SARS-CoV-2, new research suggests that its tropism may not be totally connected with ACE2 expression. Other variables, such as proteases and glycans, may have a role in determining cellular sensitivity to this viral infection [59,60,61]. A recent study revealed that HS could bind to the SARS-CoV-2 spike protein’s receptor-binding domain (RBD, the C-terminal region of the S1 subunit) and modify its shape. The fascinating potential that differences in HS and sialic acid properties could influence virus tropism prompted us to look into SARS-binding of CoV-2 to a variety of HS and sialic acid-containing oligosaccharides [62,63].

## 5. Structural Composition of SARS-CoV-2

**Figure 2:**
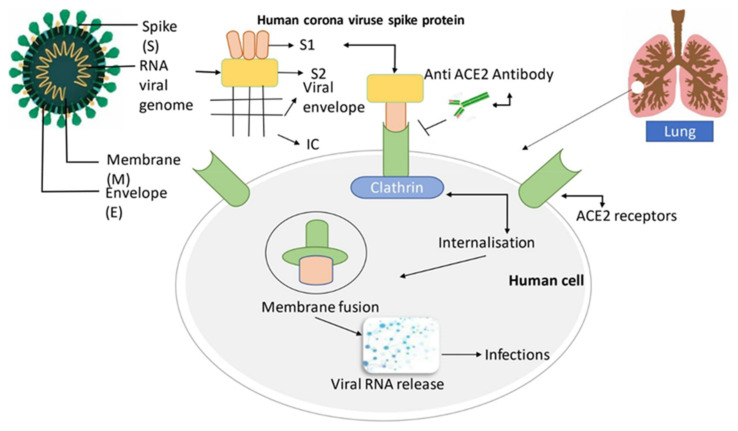
The structure of the SARS-CoV-2 virus. Clathrin is a protein that plays a vital role in the formation of coated vesicles. The S protein is an important element of the viral shape that helps the virus to connect with the host receptor cells. The S protein comprises two parts: the receptor-binding component S1 and the membrane fusion component S2; the former attaches to the human host cell’s ACE2 receptor, while the latter internalizes and forms a membrane fusion between the viral component and the ACE2 receptors. This causes the viral RNA to be released into the host cell, resulting in respiratory infection [75]. Adapted with permission from ref. [75]. Copyright 2020 Elsevier B.V.

With a genomic diameter varying from 27 to 32 kilobases in size (~125 nm or 0.125 µm), SARS-CoV-2 belongs to the biggest RNA virus family. It is an enveloping RNA virus with a single-stranded (+ssRNA) positive-sense RNA genome, with a 5′ cap shape and also a 3′ poly-A tail [64]. SARS-CoV-2 shares some characteristics with other virus infections in this family. The envelope (E), membrane (M), spike (S), and nucleocapsid protein (N) are four critical structural proteins that influence the virus’s function and shape [65]. The most important of these four proteins are N and S, the former of which aids in the optimal utilization of the capsid and the entire viral structure, and the latter of which aids in the future growth of the capsid, with the full viral framework facilitating virus attachment to host cells [66,67]. The three primary elements of the S protein are a huge ectodomain, a short intracellular tail, and also a single-pass transmembrane anchor. Those are all necessary for the host cells to be anchored. The S1 receptor attaching subunits and the S2 membrane fusion subunit are the two parts of the ectodomain (Figure 2). The clove-trimeric or crown shape in which such subunits are located gives the virus its name [68]. According to studies, SARS and SARS-CoV-2 have identical receptors within viral genomes, especially in the receptor-binding domain (RBD) and receptor-binding motif (RBM). Throughout SARS infection, the RBM of the S protein is intimately related to ACE2 in mammalian or host cells. The coronavirus’s principal targets are indeed the kidneys, lungs, and gut, while the ACE2 protein is detected in a range of human organs [69,70,71,72,73]. Although no research has established that the virus may impair men’s fertility or sexual potency because of the virus’s new nature, doctors in Wuhan have speculated that the condition may influence sperm production, a low sperm rate, and the production of male sex hormones, resulting in low libido. SARS-CoV-2 also attacks host cells through ACE2 receptors, leading to COVID-19-related pneumonia, acute myocardial damage, and long-term cardiovascular injury [74]. 

## 6. SARS-CoV-2: Suggested Mode of Action

**Figure 3:**
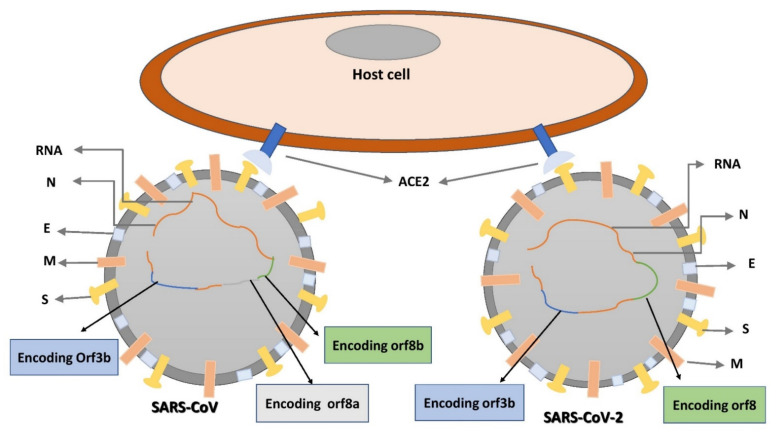
Graphical representation of the comparisons between SARS-CoV and SARS-CoV-2. N: nucleocapsid protein; E: envelope; M: membrane; S: spike.

SARS-CoV-2 shares similarities with SARS-CoV, but it does have a much higher risk of transmission and virulence; this higher rate of spread could be because of a gain of point mutations, which distinguishes this distinct virus from SARS-CoV. The 8a segment of SARS-CoV-2 is absent, the 8b and 3b segments are longer and also shorter, and the Nsp 2 and 3 proteins are changed (Figure 3) [75]. 

**Figure 4:**
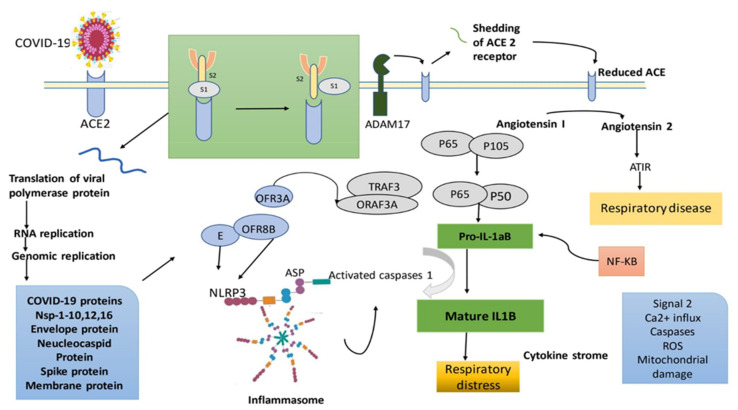
Potential mode of action of SARS- CoV-2, illustrated as SARS-CoV-2 attached to its target ACE-2. After that, the S1 and S2 molecules are cleaved, and ADAM 17 sheds ACE-2. As a consequence, the body’s level of Angiotensin II rises, resulting in respiratory discomfort. During contact, the virus fuses with the membrane and penetrates the cell, where protein synthesis and replication occur. ORF8b, ORF3a, and E proteins, and also the pathway of NF-κB, all stimulate the inflammasome path, resulting in cytokine stimulation in different ways. A cytokine storm ensues, leading in respiratory discomfort. Here, ACE2, angiotensin converting enzyme 2; NF-κB, nuclear factor- kappa B; ROS, reactive oxygen species; P65, P105, and P50 are the subunit of NF-κB transcription complex; TRAF3, TNF receptor associated factor 3; ATR maintain to genome integrity by stabilizing replication forks and by regulating cell cycle progression and DNA [75]. Adapted with permission from ref. [75]. Copyright 2020 Elsevier B.V.

SARS-CoV-2 Nsp 2 is a mutation that is thought to be linked to the virus’s capacity to be more infectious [76]. The SARS-CoV-2 orf8 and orf10 proteins are distinct. It can be helpful to learn more about the physiological functions of such proteins. Understanding the function of these proteins might be valuable. Moreover, a furin-like cleave site throughout the S protein has been revealed in novel pathogenic viruses, which is lacking in SARS-CoV but active in SARS-CoV-2. This might be the cause of SARS-CoV-2’s increased pathogenicity. SARS-CoV-2 also interacts with almost the same ACE2 receptor as SARS-CoV, but with a significantly stronger affinity; this might explain the enhanced transmission rate and ease with which it may infect other species. The S1 portion of the S protein, which has S1 just on the N terminal and S2 on the C terminal, contains RBD. The S2 domain of an S protein contains the fusion protein, an S2, an internal fusion peptide (FP), and two heptad-repeat domains surrounding the transmembrane domain (TM). SARS-CoV-2 and SARS-CoV has similar internal FPs [77]. SARS-CoV recruits proteases once it binds to the receptor and cleaves the S protein into S1 and S2 domains. S2 undergoes a structural shift as a result of the fragmentation, which is accompanied by the incorporation of the FP into the membrane and membrane fusion, allowing the virus to more readily enter the cell. ACE2 is broken and released into the additional membrane region by ADAM17 as soon as the virus penetrates the cell. Alveolar injury and higher pulmonary vascular permeability have both been associated with decreased ACE2 [78]. Once the virus’s proteins are imported into the cell, the ORF3a protein is generated, which encodes for a Ca^2+^ ion pathway identical to SARS and SARS-CoV-2. ORF3a links with TRAF3 to stimulate the nuclear factor kappa B (NF-ĸB) pathway, which causes the pro-IL-1B gene e to be transcribed [79]. ORF3a also recruits an inflammasome complex with TRAF3. NLRP3, ASC, and caspase 1 make up this complex. A secondary signal, such as Ca^2+^ influx, activation caspase, reactive oxygen species (ROS) generation, or mitochondrial injury convert pro-IL-1B to IL-B, culminating in cell proliferation. The extended ORF8b protein in SARS-CoV-2 also activates the inflammasome system via NLRP3 [80]. A cytokine storm happens because all of those channels are active, leading to the respiratory problem, which is a common sign of COVID-19. The JNK cascade, which is triggered by ORF3a, ORF3b, and ORF7a, may result in higher generation of pro-inflammatory mediators, resulting in greater lung injury (Figure 4) [81].

## 7. Therapeutic Approach against COVID-19

### 7.1. Pharmacological Drugs

#### 7.1.1. Remdesivir

Remdesivir (GS-5734) is an adenosine analog antiviral prodrug that was used to tackle the Ebola virus infection in Western Africa from December 2013 to January 2016. By attaching RNA-dependent RNA polymerase, remdesivir’s active form (GS-441524) suppresses viral RNA multiplication [68,82,83,84]. Remdesivir is an antiviral drug that works against such a range of single-stranded viruses such as RNA and includes paramyxoviruses, filoviruses, pneumoviruses, and coronaviruses, including SARS- and MERS-CoV [84,85]. Remdesivir is a prodrug that is metabolized into GS-441524, an adenine nucleotide analogue that inhibits viral RNA production. Remdesivir functions initially in the target cells, decreasing viral RNA rates in a dose-dependent manner [82], which correlates to viral load dysfunction in vitro. For the Ebola virus, SARS-, and MERS-CoV, similar modes of action of remdesivir have been established in vitro [86,87]. In cultured cells experiments, remdesivir prevents SARS-CoV-2 illness in simian Vero E6 cells infected with SARS-CoV-2 at 1.76 µM in an 90% effective concentration (EC_90_), a quantity achieved in wild monkey experiments. Remdesivir was also shown in virus-prone Huh-7 cells of human liver cancer to prevent SARS-CoV-2 infection [88]. Nowadays, remdesivir’s efficacy in preventing and treating MERS-CoV infection in nonhuman primates has been shown [89]. Patient safety data for remdesivir was obtained from a randomized, clinical study of Ebola virus treatment that was done in August 2018 in response to the Ebola virus outbreak in the Democratic Republic of Congo. A sample of 175 individuals was given remdesivir at a packed dose of 200 mg on Day 1 and then a maintenance dose of 100 mg for 13 days; nine of the people investigated had major adverse effects, demonstrating that remdesivir is a safe medicine [90]. 

#### 7.1.2. Chloroquine (CQ) and Hydroxychloroquine (HCQ)

CQ and HCQ are most commonly used as antimalarial drugs, having lysosomotropic effects. Particularly in comparison to CQ, HCQ is a less hazardous antimalarial drug made by combining chloroquine with an OH group. The effects of these antimalarial medications are widely found in the whole body, including in the lungs, according to pharmacokinetic studies [87]. They are now being researched as a potential therapy for SARS-CoV-2 because of their immunomodulating and anti-inflammatory properties [88]. Although preclinical research suggests that CQ and HCQ can reduce viral replication and may prevent COVID-19 infection, present evidence does not support their efficacy as a SARS-CoV-2 infection prophylaxis [91]. Experts recommended to utilize the CQ/HCQ regimen for SARS-CoV-2 infection prophylaxis, notably among healthcare workers who are at a higher risk of infection [92,93]. However, this opinion was refuted by data from a well-designed randomized controlled trial on 821 participants. Within four days of exposure, participants were randomly assigned to receive either HCQ or the placebo. The occurrence of novel COVID-19-related symptoms did not differ significantly between the two groups (11.8% versus 14.3%; *P* = 0.35) [94]. Another study also showed differences between CQ/HCQ and the placebo, but leading without showing any significant effect againt SARS-CoV-2 [95].

#### 7.1.3. Lopinavir/Ritonavir

Lopinavir is an extremely effective blocker of HIV protease, which is necessary for intracellular HIV replication. It was created in 1998 to combat HIV tolerance of the protease inhibitor ritonavir, which is produced by a variation of valine at position 82 (Val 82) in HIV protease’s active site in reaction to ritonavir treatment. Because lopinavir decomposition is inhibited by ritonavir, concomitant oral treatment of ritonavir and lopinavir exceeded the in vitro antiviral half maximal effective concentration (EC_50_) of lopinavir in monkeys, dog, and rat plasma by over 50-fold after 8 h [96]. As a result, when taken in combination with other antiretroviral drugs, the combination of lopinavir and ritonavir has indeed been acknowledged as an excellent orally administered medicine in the treatment of HIV-infected persons [97,98]. 

#### 7.1.4. Tocilizumab

Tocilizumab is a humanized monoclonal antibody that targets the interleukin-6 receptor (IL-6R) and has been allowed by the FDA to treat rheumatoid arthritis. In critically sick COVID-19, the cytokine release storm (CRS) has been mediated by IL-6. As a result, it has been recommended as a treatment for such patients [73,99]. In China and Italy, tocilizumab has been used as an immuno-suppressive medication in COVID-19 patients with impressive results. In China, individuals with COVID-19 who were given tocilizumab, showed massive improvement, implying that tocilizumab could be very effective in curing people with acute disease [100,101,102].

#### 7.1.5. Favipiravir

Toyama Chemical, Japan, developed favipiravir that inhibits the RNA-dependent RNA polymerase (RdRp) of RNA viruses and produces lethal RNA transversion mutations, resulting in a nonviable viral phenotype [103,104,105]. Favipiravir inhibits the replication of a wide range of RNA viruses, such as influenza A, Ebola, and Lassa viruses [106]. The favipiravir-treated individuals had a substantially superior therapeutic response, with quicker viral clearance and a higher incidence of improvement in chest imaging. Based on these promising results, China’s National Medical Products Administration has authorized favipiravir as the country’s first anti-COVID-19 drug [107].

#### 7.1.6. Umifenovir

**Figure 5:**
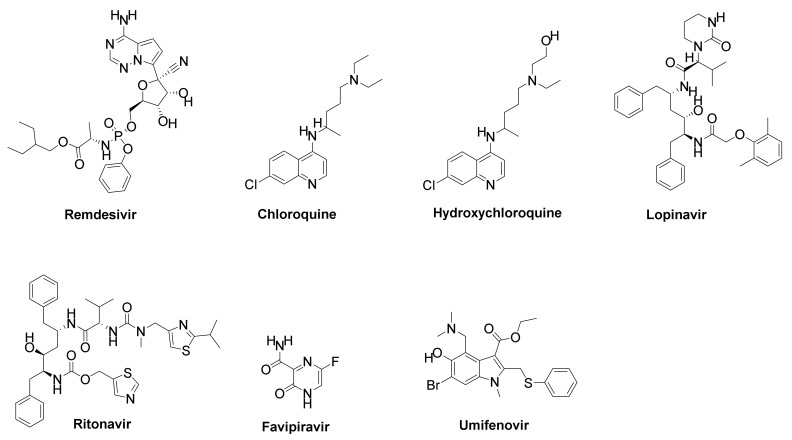
The structures of some effective COVID-19 treatment pharmacological drugs.

Umifenovir is a small indole-derivate molecule that protects against viral infection by inhibiting clathrin-mediated endocytosis, which hinders membrane fusion between the viral particle and the host cell’s cytoplasmic membrane (Figure 5). In Russia and China, umifnovir is approved for the treatment and control of influenza A and B viruses, as well as for many other respiratory pathogens [106,108,109,110]. Around 36 COVID-19 patients had taken 400 mg umifenovir three times per day for nine days in a clinical pilot project in Wuhan, China, with 31 uncontrolled COVID-19 patients taking part as a control condition. When compared with the control group, umifenovir therapy was found to have a higher proclivity for reducing viral load as determined by RT-PCR, and also a reduced fatality rate of 0% vs. 16% [111]. Table 1 illustrates some combinational drugs that can act as potential targets for COVID-19.

### 7.2. Natural Products for COVID-19 Treatment

Nonetheless, the expensive healthcare expenditure, lack of availability, unprecedented drug side effects, and ethical considerations regarding convalescent plasma therapy (CPT) make them difficult to execute globally. Furthermore, the problem can be made worse if the virus evolves into drug-resistant mutants, rendering antiviral medications worthless because most of them target specific viral proteins [117]. As a result, we can search for new therapies using natural products. Some of the potential natural products antiviral medications utilized for the management and prophylaxis of COVID-19 are reviewed in the following sections of this review (Table 2).

#### 7.2.1. Alkaloids Derivatives

Homoharringtonine is a cytotoxic alkaloid isolated in the *Cephalotaxus hainanensis* medicinal plant. It has been approved by the FDA as a treatment for resistant chronic myeloid leukemia. Homoharringtonine has the smallest half maximal inhibitory concentration (IC_50_) and has significant antiviral efficacy against a number of human and animal coronaviruses [144]. The antiallergic, antimalarial, antibacterial, and antiviral effects of isatin (1H-indole-2,3-dione), an oxidizing indole derivative, have been found in nature, including *Isatis tinctoria* and *Calanthe discolor*. SARS-CoV 3C-like protease (3CL^pro^) was suppressed in tiny doses by isatin derivatives [145,146,147]. Rhinovirus and SARS-CoV have identical protease architectures. In coronavirus-infected swine testicular cells, *Tylophora indica*’s tylophorine and 7-methoxycryptopleurine were found to limit viral prolification. In this investigation, 7-methoxycryptopleurine IC_50_:20 nM was found to be more effective than tylophorine (IC_50_:58 nM). In previous studies, tylophorine was demonstrated to affect viral RNA proliferation and cellular JAK2-mediated dominating nuclear factor kappa B (NF-ĸB) activation in CoV at doses of 0–1000 nM [148,149].

As per the MTS testing for virus-induced cytopathic impact, *Lycoris radiata* extract has high antiviral efficacy against SARS-CoV. The active ingredient in this extraction is lycorine, an alkaloid with an EC_50_ of 15.7 1.2 nM, showing antiviral action. These results indicate that lycorine could be a promising candidate for developing novel antiviral medicines. In vitro, lycorine also inhibited the reproduction of coronaviruses such as HCoV-OC43 (EC_50_:0.15 µM), MERS-CoV (EC_50_:1.63 µM), and HCoV-NL63 (EC_50_:0.47 µM), according to another study [150,151].

Anticancer, anti-inflammatory, and antioxidant effects are reported for bisbenzylisoquinoline alkaloids found in the roots of *Stephania tetrandra*. The main active *S. tetrandra* alkaloids that have also showed antiviral efficacy towards human coronavirus-OC43 (HCoV-OC43) (betacoronavirus 1) disease, are tetrandrine (IC_50_: 14.51 µM), fangchinoline (IC_50_: 12.40 µM), and cepharanthine (IC_50_: 10.54 µM). Emetine, also an alkaloid, is the active ingredient of *Carapichea ipecacuanha* roots that contains anti-protozoal and vomiting medicines properties. Many coronaviruses, including HCoV-OC43 (EC_50_: 0.30 µM), MERS-CoV (EC_50_: 0.34 µM), and human coronavirus-NL63 (HCoV-NL63) (alphacoronavirus), were suppressed in vitro by emetine (EC_50_: 1.43 µM). Additionally, emetine can protect host cells against MERS-CoV infection [152]. Many coronaviruses, such as HCoV-OC43 (EC_50_: 0.30 µM), MERS-CoV (EC_50_: 0.34 µM), and HCoV-NL63, were suppressed in vitro by emetine (EC_50_: 1.43 µM). Moreover, emetine can protect host cells from MERS-CoV infection [152].

#### 7.2.2. Polyphenols and Flavonoids Derivatives

Twelve geranylated flavonoids, including five new compounds 8 (tomentin A–E) (2.39–2.43) identified from the traditional Chinese medicinal (TCM) plant *Paulownia tomentosa* (Thunb.) Steud., inhibited SARS Papain-Like Protease (PL^pro^) in a mixed-type manner, with IC_50_ values ranging from 5.0 to 14.4 µM. Tomentin A, B, and E were revealed to be the most effective PL^pro^ inhibitors in this group, with IC_50_ values of 6.2, 6.1, and 5.0 µM, respectively. Each of these novel compounds with the dihydro-2H-pyran moiety inhibited more effectively than their parent compounds [130]. 

Similarly, six flavonoids isolated from *Cullen corylifolium* (L.) Medik. seeds (bavachinin, neobavaisoflavone, isobavachalcone, 4′-*O*-methylbavachalcone, psoralidin, and corylifol A) showed mixed-type inhibition against SARS-CoV PL^pro^, with IC_50_ values ranging from 4.2 to 38.4 µM [153].

Bioflavonoids, such as amentoflavone isolated from *Torreya nucifera*, have been shown to have noncompetitive 3CL^pro^ inhibitory action with low micromolar IC_50_ values. The most powerful inhibitor (IC_50_ = 8.3 µM) was found to be amentoflavone (2.6), which was much more potent than the parent chemical apigenin (IC_50_ = 280.8 µM). Other apigenin-containing flavones, such as luteolin (2.23) (IC_50_ = 20.2 µM) and quercetin (2.29) (IC_50_ = 23.8 µM), similarly inhibited 3CL^pro^ more than the parent, showing that the apigenin moiety at position C-3′ of flavones is crucial for effectiveness. Honeysuckle’s primary flavonoid, luteolin, is a component of *Lianhua qingwen*, a TCM for COVID-19 [154].

Baicalin is a glycosylated flavonoid derived from *S. baicalensis* that displays antiviral activity against by the fRhK-4 cell line’s prototype virus (EC_50_ 12.5 µg/mL). At concentrations of 0.1 µM and higher, baicalein reduced the cell damage caused by SARS-CoV-2 and enhanced the morphology of Vero E6 cells. Oral administration of 200 mg/kg crystal form β of baicalein to rats resulted in an effective concentration. Baicalein also reduced body weight loss, virus multiplication, and lung tissue lesions in hACE2 transgenic mice treated with SARS-CoV-2 [130,155,156]. 

Polyphenols obtained from *Angelica keiskei* have chalcones with a C-5 prenyl that exhibit strong inhibitory action against 3CL^pro^ and PL^pro^ in vitro. Noncompetitive inhibition of PL^pro^ was shown by alkylated chalcones, with the most effective compounds being xanthoangelol E (IC_50_: 1.2 µM) and xanthoangelol F (IC_50_: 5.6 µM). According to the analysis of SAR, the perhydroxyl member of a chalcone is an alkylated chalcone with a stronger inhibitory effect [157].

Resveratrol is a stilbenoid found in *Vitis vinifera*, *Vaccinium macrocarpon*, and *Polygonum cuspidatum*, among other plants. Hepatoprotective, cardioprotective, neuroprotective, antiinflammatory, and antibacterial properties are only some of the pharmacological and therapeutic benefits of resveratrol. In vitro, resveratrol significantly inhibit MERS-CoV proliferation and reduced MERS-CoV infection. As a consequence, resveratrol is an important anti-MERS medication and could be a viable SARS-CoV2 antiviral [158,159].

The structure–activity relationship (SAR analysis) of quercetin-3-galactoside and its replaced analogues exposes (1) that the 4 OH groups upon a quercetin moiety are important for biological action; (2) that trying to remove the 7-OH decreases the 3CL^pro^ inhibitory effect; (3) that the sugar moiety is important for action; and (4) that sugar alterations have no influence on inhibitor efficacy [160].

Myricetin and scutellarein are obtained from *Nigella sativa*, which have been reported in many studies. At concentrations of 0.01–10 µM, myricetin and scutellarein inhibit SARS-CoV 3CL^pro^. Broussochalcone B, broussochalcone A, 4-hydroxyisolonchocarpin, papyriflavonol A,4,7-trihydroxyflavane, kazinol A, kazinol B, broussoflavan A, kazinol F, and kazinol J are all obtained from *Broussonetia papyrifera*, which are also responsible for inhibiting SARS-CoV [126,161,162].

#### 7.2.3. Terpenoid Derivatives

Quinone-methide triterpenes are a kind of terpene found solely in the Celastraceae family of plants, such as *Tripterygium regelii*. With an IC_50_ of 2.6–10.3 µM, these compounds demonstrated modest inhibitory action towards 3CL^pro^. The presence of a quinone-methide molecule, according to SAR analysis, plays a substantial role in 3CL^pro^ inhibition [163]. 

Saikosaponins are the main pharmacological active triterpenoids, usually as glucosides, found from TCM such as *Bupleurum* spp., *Heteromorpha* spp., and *Scrophularia scorodonia*, with antiviral and immunomodulatory potential [164]. Four saikosaponins, namely, saikosaponin A, B2, C, and D (5–25 M/L), demonstrate action towards human coronavirus-229E (CoV-229E) (alphacoronavirus) with EC_50_ values of 8.6, 1.7, 19.9, and 13.2 µM, respectfully; saikosaponin B2 suppressed viral adherence and penetration stages [165].

Triterpenoids and 3-friedelanol obtained from *Euphorbia neriifolia* leaves were tested in vitro for anti-HCoV efficacy in 2012. 3-Friedelanol with a triterpenoid showed through screening a more potential antimicrobial action and increased cellular viability after incubation with HCoV. Furthermore, 3β-fridelanol showed strong inhibitory action towards 3CL^pro^ [166,167].

*Glycyrrhiza glabra* and glycyrrhizin, its active ingredient, have antiviral action against a variety of viruses, including hepatitis A, B, C, varicella-zoster, HIV, and herpes simplex type-1 [168].

*Salvia miltiorrhiza* produces tanshinones with an abietane diterpene structure. Tanshinones have several biological actions, including anti-inflammatory, cardiovascular, and anti-tumor properties. These compounds preferentially block the SARS-CoV 3CL^pro^ and PL^pro^ enzymes, and their effectiveness varies depending mostly on enzyme subtype. Some tanshinones inhibit PL^pro^ more potently (IC_50_ varying from 0.8 to 30.0 µM) [169].

**Figure 6:**
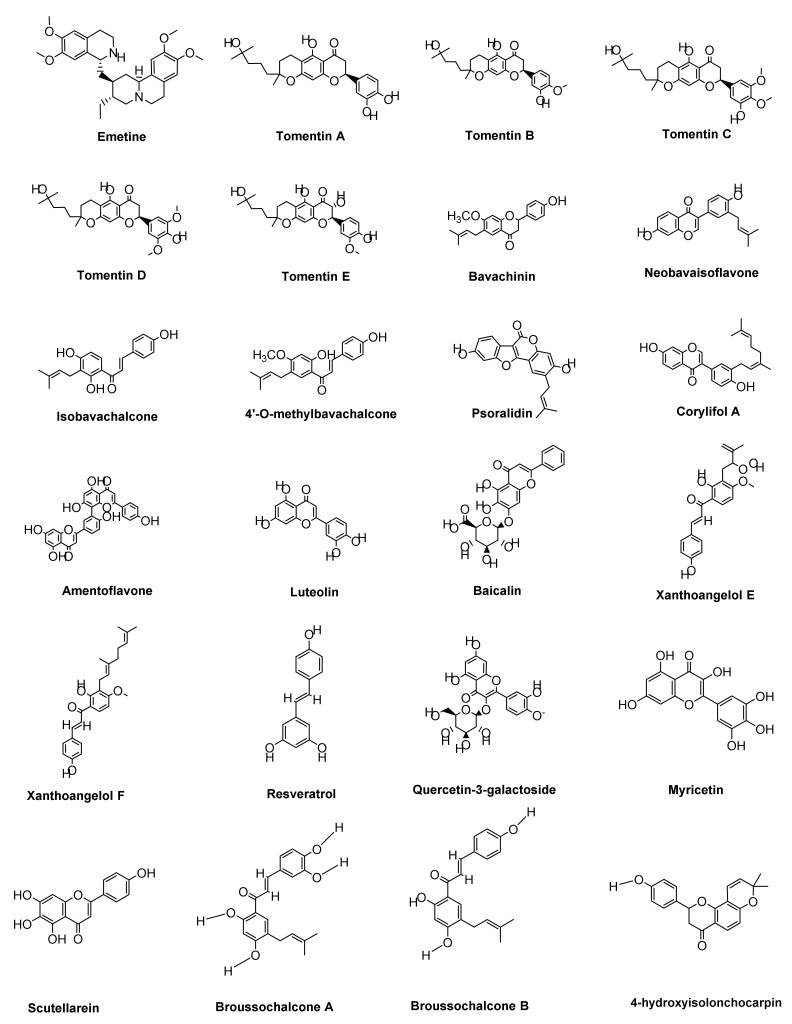

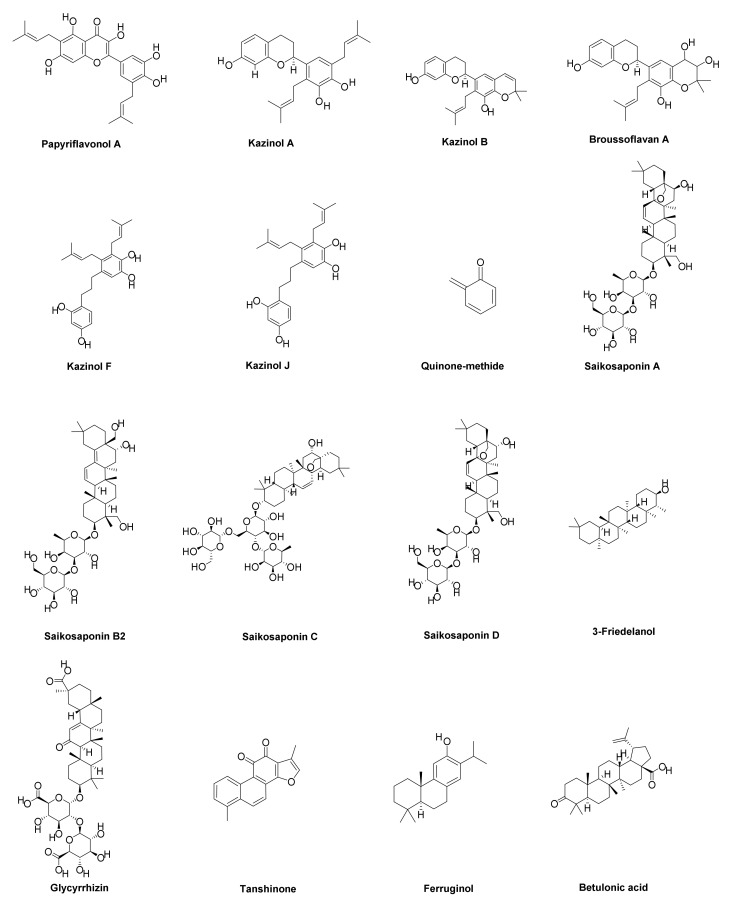

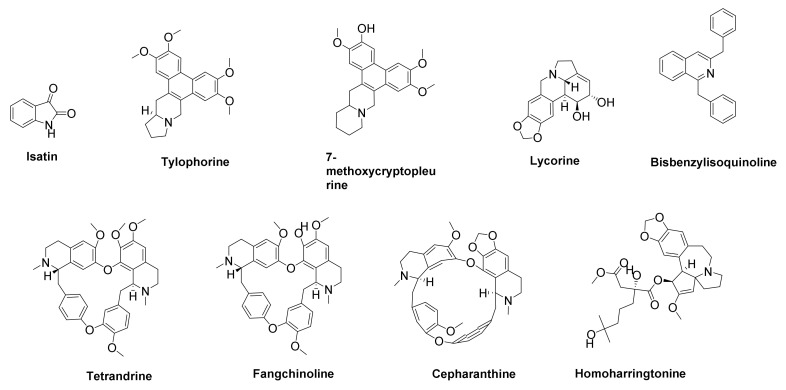
The structures of some effective COVID-19 treatment natural products (alkaloids, polyphenols, flavonoids, and terpenoids derivatives).

Ferruginol, a natural phenol with a terpenoid substructure derived from *Sequoia sempervirens*, has been shown to have anticancer effects in humans with colon, breast, and lung malignancies. Furthermore, at 0–80 µM, ferruginol, betulonic acid, betulinic acid, hinokinin, savinin, and curcumin are some of the compounds found in turmeric, which reduced SARS-CoV replication substantially [136,170]. The structures of some effective COVID-19 treatment natural products are shown in Figure 6.

#### 7.2.4. Miscellaneous Compounds

Sivestrol, a natural substance derived from the fruit of *Aglaia foveolata*, has been demonstrated to exhibit highly potent in vitro cytotoxic activity against a number of human cancer cell lines. Furthermore, at doses of 0.6–2 µM, this drug inhibited nondependent viral mRNA synthesis of HCoV-229E with an IC_50_ of 40 nM [171,172].

One of the promising antibiotic treatments against SARS-CoV is valinomycin with a cyclododecadepsipeptide architecture, which was discovered in *Streptomyces tsusimaensis* and has minimum cytotoxicity and great efficiency against CoV [173].

Phycocyanin, lutein, polysaccharides, vitamins, and other phenolics were found to have antibacterial, anticancer, anti-inammatory, and other important pharmacological effects in marine microalgae belonging to the phyla Rhodophyta and Phaeophyta [120]. Hirata et al. [174] investigated the antiviral and antioxidative properties of phycocyanobilins, a type of tetrapyrrole chromophores found in some marine cyanobacteria. In silico molecular docking tests conducted by Pendyala and Patras in 2020 revealed that phycocyanobilins had a significant binding affinity for the SARS-CoV-2 main protease (M^pro^) and RdRp. Lectins are a type of molecule with a strong affinity for carbs. Griffithsin, a lectin produced from red algae, was investigated for its possible application, and tests have revealed that it has antiviral action against human immunodeficiency virus-1 (HIV-1) and hepatitis C [175,176,177]. A recent in vitro study by Millet et al. [178] revealed that griffithsin had inhibitory action against MERS-CoV. Esculetin ethyl ester from *Axinella* cf. *corrugate*, a marine sponge, demonstrated a high interaction with SARS-CoV-2 protease and could be employed as an anti-COVID-19 drug [179]. Carrageenans, a type of sulphated polysaccharide found in the sea, are considered to be virus inhibitors. They work by preventing the virus from attaching and then being internalized. As a result, Nagle et al. [180] hypothesized that these compounds could be used as coating materials on hygienic products to inhibit COVID-19 infection. In silico analyses have recently contributed in the identification of potential lead compounds for therapeutic development against the COVID-19 pandemic. In a molecular dynamic research, Khan et al. [181] reported four effective SARS-CoV-2 M^pro^ inhibitors from marine sources (fostularin 3,1-hexadecoxypropane-1, 2-diol, palmitoleic acid, 15 alphamethoxypuupehenol, and puupehedione) that can be used to disrupt the viral life cycle in the host.

Lactoferrin (LF), a transferrin-family glycoprotein found in many different of human secretions, is known to bind and transport iron and to play a key role in iron homeostasis regulation. In vitro studies on human intestinal, liver, and T cell lines revealed that LF possesses promising antiviral and antibacterial properties, as well as anti-inflammatory and immune-modulating properties [182,183,184,185]. Many investigations have shown that LF has potent antiviral activity against viruses such as hepatitis C virus, herpes simplex virus, human immunodeficiency virus, poliovirus, and rotavirus, with EC_50_ values in the micromolar range in vitro [186,187]. The LF antiviral action is most evident in the early stages of infection, when it prevents viral particles from entering host cells by binding directly to them or inhibiting the virus receptor or co-receptor on the host cell. Moreover, LF can inhibit some viruses from internalizing, including the SARS pseudovirus, by binding to cell-surface HSPGs, which have been demonstrated to be required co-factors for SARS-CoV-2 infection [188,189,190]. Furthermore, LF has been found to prevent the entry of murine coronavirus and human coronaviruses such as hCoV-NL63, which are closely related to SARS-CoV-2 [191,192]. SARS-CoV-2 uses endocytosis as a cell-entrance mechanism, and LF has been shown to preferentially suppress cathepsin L, a lysosomal peptidase important for endocytosis. The immunomodulatory and anti-inflammatory properties of LF are another essential component of its bioactivity [192,193,194,195]. In experimental settings simulating sepsis, LF was shown to lower IL6 and tumor necrosis factor-alpha (TNF-α). Therefore, it is feasible that LF can control the hyperactive immunological and inflammatory response to SARS-CoV-2, which can lead to acute respiratory distress and death in some individuals. Overall, LF has the potential to be a non-toxic health supplement that can be used to prevent infection as well as a supplementary treatment for those who have COVID-19 [26,196,197].

Vitamin C is a water-soluble vitamin with antioxidant effects that supports the epithelial barrier against pathogen entrance and the cellular functioning of the innate and adaptive immune systems in the immune system. Environmental factors, such as air pollution, and the presence of diseases, such as type 2 diabetes, can affect vitamin C levels in the body [198]. Vitamin C deficiency affects the older population in particular because chronic or acute disorders are frequent in this group, and aging is linked to lower vitamin C levels [199,200,201]. Furthermore, vitamin C may control the cytokine storm, which is characterized by elevated levels of the pro-inflammatory cytokine IL-6, increasing the risk of respiratory failure necessitating mechanical ventilation in COVID-19 patients. Pretreatment with vitamin C, according to an in vivo study involving 12 healthy males, can lower the amounts of IL-6 generated by the vasoconstrictor endothelin-1 (ET-1), lowering vascular dysfunction. Furthermore, elevated ET-1 expression has been linked to pneumonia, pulmonary hypertension, interstitial lung fibrosis, and acute respiratory distress syndrome (ARDS) [89,202,203,204]. 

In pulmonary epithelial cells, the vitamin D receptor (VDR) is present. When VDR is activated, it produces defensins and catelicidins, peptides that have antiviral activity either directly or through immunological regulation. The decreased antiviral immune response in COVID-19 patients during vitamin D shortage may be attributed to a reduction in LL37 levels, an antimicrobial peptide generated from catelicidin [205,206]. Vitamin D may also help to reduce the severity of inflammatory reactions by inhibiting pro-inflammatory cytokines such TNF-α and IL-6, which are involved in the development of cytokine storm in COVID-19-related ARDS [207,208].

Zinc’s immunomodulatory and antiviral properties have made it and its ionophores potential COVID-19 targets [209,210]. Zinc is crucial for immune system integrity, and it plays a key role in cell maintenance, development, and activation throughout innate and adaptive immunological responses. It also helps to maintain the integrity of epithelial barriers, which are necessary for organism defense and pathogen prevention [211,212,213]. Zinc can regulate T cell growth and activity, thus decreasing the cytokine storm, which is accompanied by significant amounts of pro-inflammatory cytokines and chemokines that cause systemic immune response dysfunction, resulting in ARDS or multiple organ failure [214,215]. Natural killer (NK) cells and cytolytic T cells, both of which are important in the elimination of viruses, bacteria, and tumor cells, are both affected by zinc deficiency [216,217]. Zinc’s direct antiviral activity is another vital role, making it necessary for the immunological response to viral infection. Increased intracellular concentrations of this mineral can inhibit viral polyprotein processing and limit the replication of a number of RNA viruses [218,219,220]. Zinc can also improve interferon (IFN) cytokine signaling against RNA viruses and reduce ACE2 activity, which is required for SARS-CoV-2 entrance into host cells [221,222]. 

## 8. Some Drawbacks of Antiviral Drugs on Human Body

### 8.1. Remdesivir

Researchers noted that remdesivir had certain negative impacts on the human body during a clinical study against SARS-CoV-2, such as improved productivity of liver enzymes, which might be responsible for liver dysfunction [223]. In recent times, experts in the United States documented many negative remdesivir impacts in individuals hospitalized with COVID-19. Moreover, remdesivir has been linked to an increased risk of allergic reactions, hypotension, breathing problems, as well as other human body anomalies in individuals with COVID-19, according to many study investigations. Researchers employed the first study to look at the efficacy of 5 or 10 days of remdesivir therapy for individuals with moderate bacterial meningitis. In the therapy group, vomiting, metabolic alkalosis, and migraine were more prevalent than customary [91,224].

### 8.2. Chloroquine (CQ) and Hydroxychloroquine (HCQ)

CQ can cause digestive side effects such as stomach pain, vomiting, sickness, and feces. CQ causes a variety of cardiovascular issues, including cardiac failure, lowers the blood pressure, a reduction in cardiac function, and dilatation. Finally, it has the potential to stop potassium from entering cells and has an influence on the chloride channels found in cardiac myocytes. CQ and HCQ can produce arrhythmias in COVID-19 patients, which can be decreased by mixing them with other medicinal medications, such as the antibiotic azithromycin, which has comparable cardiac actions. In other investigations, substantial rhythm problems have been linked to using CQ or HCQ, particularly in high dosages or in combination with the antibiotic azithromycin. It was also recognized that cardiac adverse effects were more common in women than in males. Heat transfer problems had the most common adverse effects, with ventricular hypertrophy, hypokinesia, and valve malfunction all being observed [225,226].

### 8.3. Lopinavir/Ritonavir

Patients who were given lopinavir/ritonavir experienced greater digestive problems as well as other comorbidities, which hampered their healing. In the lopinavir–ritonavir trial group, gastrointestinal problems were so much more prevalent in individuals with COVID-19 [227]. A clinical trial of 90 patients showed that about 14% receiving lopinavir–ritonavir were unable to conclude the 14-day regimen. Migraine, stomach discomfort, bloating, malnutrition, and anomalous stools were the most common gastrointestinal negative effects [228].

### 8.4. Tocilizumab

In humans, tocilizumab has a number of negative health consequences, including upper respiratory infection, migraine, nasopharyngitis, site of injection response, and increased blood pressure [229].

### 8.5. Favipiravir

Reduced locomotor activity, anemia, nausea, weight loss, enhanced demyelination in hepatocytes, and elevated blood concentrations of liver function enzymes are all side effects of favipiravir. It can potentially cause birth defects; thus, it should not be taken during pregnancy [230,231].

### 8.6. Azithromycin

It has been confirmed that azithromycin is the cause of adverse effects, including nausea and dizziness. Nonetheless, treating COVID-19 patients with azithromycin and hydroxychloroquine has resulted in very serious complications, including the risk of mortality from abrupt heart failure [232,233,234]. 

## 9. Conclusions and Future Perspectives

COVID-19 is a life-threatening infection. It has sparked the interest of everyone on the planet, irrespective of age or educational level. The virus’s effects have ceased all business, education, and travel operations, as well as disrupting people’s daily lives across the world. Everyone who has been afflicted is hoping for a quick scientific reaction to put an end to the pandemic and lessen its devastating consequences. Unfortunately, developing a new drug is protracted, and the development of a new drug is improbable in a pandemic situation where an effective therapy is required right away. Natural products have been used for many years to treat viral infections and stimulate the immunological response of the host. Natural products have shown to be effective in past coronavirus illnesses, such as SARS and MERS; thus, natural products may be beneficial and provide hope during this new outbreak. In the combat against viruses, natural compounds can be used as both preventative and therapeutic agents. The following ways may be valuable in increasing and supporting research projects on COVID-19 treatment and prevention using natural products: more investigation on the use of natural chemicals as anti-coronavirus agents; quality-assurance studies for herbal extracts for use as immune-boosting medicines should be standardized; find novel targets for battling coronavirus; investigate the pharmacokinetics, pharmacodynamics, and toxicities of purified natural compounds; and expand new promiscuous drugs using SAR analysis and scientific in vivo and clinical studies.

## Figures and Tables

**Table 1:** Combinational drugs as potential COVID-19 treatment.

Drugs	Targeted Virions Infection	Targeted Virions Modes	References
HCQ and Itazoxanide	SARS-CoV-2	Used as adjuvant treatment in COVID-19	[111]
Darunavir and Umifenovir	SARS-CoV-2	Inhibition of viral replication reduces viral load	[112]
CQ and HCQ	SARS-CoV-2	Reduces viral loads in the lungs and increases pulmonary activity	[113]
Interferon beta, Lopinavir and ritonavir	MERS-CoV and SARS-CoV-2	The viral load was reduced little, and pulmonary function improved slightly	[114]
Ribavirin and Interferon-α	SARS-CoV-2	ARDS was reduced, as was mortality	[115]
Camostat mesilate HCQ	SARS-CoV-2	Blockers of the angiotensin receptor and inhibitors of the serine protease of the host cell	[116]

**Table 2:** List of some natural products, their classification, and possible mechanism of action.

Sl. No.	Name of Natural Product	Class of the Compound	Source	Biological Efficacy	References
1	Daurisoline	Alkaloid	*Rhizoma menispermi*	Increased endolysosomal pH, lowered active cathepsin levels, and impaired V-type ATPase activity (EC of 10 µM)	[118,119]
2	Dauricine	Alkaloid	*Rhizoma menispermi*	Increased endolysosomal pH, lowered active cathepsin levels, and impaired V-type ATPase activity (EC of 10 µM)	[120]
3	Tetrandrine	Alkaloid	*Stephania tetrandra*	Increased endolysosomal pH in a concentrationdependent manner (EC of 1–10 µM)	[121,122]
4	Luteolin	Flavonoid	*Rhodiola kirilowii*	IC_50_ = 4.5 µM	[123,124]
5	Quercetin	Flavonoid	*Allium cepa*	IC_50_ = 83.4 µM	[125]
6	Kazinol A	Flavonoid	*Broussonetia papyrifera*	IC_50_ = 84.8 µM	[126]
7	Kazinol F	Biphenyl propanoids	*Broussonetia papyrifera*	IC_50_ = 43.3 µM	[127]
8	Kazinol J	Biphenyl propanoids	*Broussonetia papyrifera*	IC_50_ = 64.2 µM	[127]
9	Kaempferol	Flavonoid	*Zingiber officinale*	IC_50_ =16.3 µM	[128]
10	Neobavaisoflavone	Flavonoid	*Psoralea corylifolia*	IC_50_ = 18.3 µM	[129]
11	Papyriflavonol A	Flavonoid	*Broussonetia papyrifera*	IC_50_ = 3.7 µM	[126]
12	Psoralidin	Flavonoid	*Psoralea corylifolia*	IC_50_ = 4.2 µM	[129]
13	Tomentin A	Flavonoid	*Paulownia tomentosa*	IC_50_ = 6.2 µM	[130]
14	Tomentin B	Flavonoid	*Paulownia tomentosa*	IC_50_ = 6.1 µM	[130]
15	Tomentin C	Flavonoid	*Paulownia tomentosa*	IC_50_ = 11.6 µM	[130]
16	Tomentin D	Flavonoid	*Paulownia tomentosa*	IC_50_ = 12.5 µM	[130]
17	Tomentin E	Flavonoid	*Paulownia tomentosa*	IC_50_ = 5.0 µM	[130]
18	Catechin	Flavonoid	*Camellia sinensis*	Elevated Zn^2+^ level (2-fold increase at EC of 50 µM)	[131]
19	Epigallocatechin-3-gallate (EGCG)	Flavonoid	*Camellia sinensis*	Elevated intracellular Zn^2+^ level (2-fold increase at EC of 50 µM)	[131]
20	Rutin	Flavonoid glycoside	*Morus alba*	Elevated intracellular Zn^2+^ level (4-fold increase at EC of 50 µM)	[131]
21	Apigenin	Flavonoid	*Adinandra nitida*	30.3% suppression at EC of 500 µg/mL	[132,133]
22	Camellianin A	Flavonoid	*Adinandra nitida*	30.2% suppression at EC of 500 µg/mL	[132,133]
23	Camellianin B	Flavonoid	*Adinandra nitida*	40.7% suppression at EC of 500 µg/mL	[132,133]
24	Taxifolin	Flavonoid	*Coreopsis tinctoria*	IC_50_ = 145.7 µM	[134]
25	Myrtenal	Terpene	*Elettaria cardamomum*	Suppressed the action of V-type ATPase and reduced endolysosomal acidification (EC of 100 µM)	[135]
26	Pulsatilla saponin D	Triterpenoid saponin	*Pulsatilla chinensis*	Increased endolysosomal pH and downregulated cathepsins (EC of 1.25 µM)	[88]
27	Betulinic acid	Terpenoid	*Breynia fruticose*	IC_50_ = 10.0 µM	[136,137]
28	Leelamine	Terpene	*Pinus sylvestris*	Decreased endolysosomal acidity and suppressed cellular endocytosis (EC of 3 µM)	[138]
29	Curcumin	Polyphenol	*Curcuma longa*	IC_50_ = 5.7 µM	[137]
30	Caffeic acid	Phenolic acid	*Ocimum basilicum*	Elevated intracellular Zn^2+^ level (3-fold increase at EC of 50 µM)	[131]
31	Catechol	Phenol	*Allium cepa*	Elevated intracellular Zn^2+^ level (2-fold increase at EC of 50 µM)	[131]
32	Gallic acid	Phenolic acid	*Syzygium aromaticum*	Elevated intracellular Zn^2+^ level (4-fold increase at EC of 50 µM)	[131]
33	Resveratrol	Polyphenol	*Vitis vinifera*	Elevated intracellular Zn^2+^ level (7.5-fold increase at EC of 10 µM)	[139]
34	Methyl gallate	Phenolic acid	*Tamarix hohenackeri*	35.7% suppression at EC of 20 mg/mL	[140]
35	Tannic acid	Phenolic acid	*Camellia sinensis*	IC_50_ = 5.7 µM	[141]
36	Pd-C-I	Coumarin	*Angelica decursiva*	IC_50_ = 6.8 µM	[142]
37	Pd-C-II	Coumarin	*Angelica decursiva*	IC_50_ = 12.4 µM	[142]
38	Pd-C-III	Coumarin	*Angelica decursiva*	IC_50_ = 15.3 µM	[142]
39	Isorutarine	Coumarin	*Angelica decursiva*	IC_50_ = 68.4 µM	[142]
40	Ampleopsin C	Stilbenoid	*Vitis thunbergiivar*	IC_50_ = 18.4 µM	[143]

## Data Availability

Not applicable.
